# Honey Bee as Alternative Medicine to Treat Eleven Multidrug-Resistant Bacteria Causing Urinary Tract Infection during Pregnancy

**DOI:** 10.3390/scipharm86020014

**Published:** 2018-04-13

**Authors:** Mabrouka Bouacha, Hayette Ayed, Nedjoud Grara

**Affiliations:** 1 Laboratory of Biochemistry and Microbiology, Department of Biochemistry, Faculty of Sciences, University of Badji Mokhtar, 23000 Annaba, Algeria; 2 Department of Biology, Faculty of Biology, University 8 May 1945, 24000 Guelma, Algeria; rymadafri@yahoo.fr (H.A.); grara120@yahoo.fr (N.G.)

**Keywords:** honey bee, urinary tract infections, pregnant women, antibacterial activity, alternative medicine

## Abstract

Medicinal benefits of honey bee have been recognized in the medical community since ancient times as a remedy for many diseases and infections. This study aimed to investigate the *in vitro* susceptibility of 11 multidrug-resistant bacterial strains, isolated from urinary tract infections of pregnant women, to six honey samples collected from different localities in the east of Algeria. The evaluation of the antibacterial activity was performed by the well method followed by the broth dilution method using two-fold dilutions of each honey sample ranging from 2.5 to 80% (*w/v*). The results obtained in this study revealed that all tested honeys exhibited potent antibacterial activity against the tested strains. The diameters of inhibition ranged from 19.67 to 53.33 mm, with minimum inhibitory concentrations (MICs) ranging from 2.5 to 40% (*w/v*) and minimum bactericidal concentration (MBCs) varied between 2.5 and 80% (*w/v*). Gram-positive bacteria were found to be more susceptible than Gram-negative bacteria with diameters ranging from 43.33 to 53.33 mm; MIC and MBC values ranged from 2.5 to 5% (*w/v*). The *P. aeruginosa* strain was found to be less susceptible than other strains with inhibitory diameters ranging from 19.67 to 27.33 mm; MICs ranged from 20 to 40% and MBCs ranged from 20 to 80% (*w/v*). This contribution has provided a broad overview of the antibacterial activity of Algerian honey and shown that honey bee has great potential for therapeutic use as an alternative therapy for urinary tract infection treatment which is safe and efficient during pregnancy.

## 1. Introduction

Urinary tract infections (UTIs) are among the most common bacterial infections, affecting approximately 150 million people worldwide each year [1]. UTIs are most frequently caused by uropathogens from fecal flora (i.e., *Escherichia coli*) that ascend the urethra to infect the bladder [2].

UTIs are considered as a serious public health burden and significantly disturb the quality of life of affected persons. UTIs are accompanied by complications such as pyelonephritis and bacteraemia which, if not treated carefully, may lead to development of permanent renal damage and significant mortality [3].

These infections can affect both men and women of all ages; however, women are more likely to experience this infection than men [4]. It was estimated that around 11% of women report at least one physician-diagnosed UTI per year and 20–30% report multiple recurrences (rUTI). This is mostly due to their shorter female urethral length compared with male urethra as a result of an easier point of access for bacterial pathogens.

On the other hand, during pregnancy, the urinary tract of women undergoes anatomical and physiological changes that can result in symptoms and conditions affecting both the mother and the fetus [5]. The hormonal and anatomo-physiological changes facilitate the growth and dissemination of bacteria in the maternal urinary tract [4,5]. They are mostly limited to the lower urinary tract and often arise from a single type of bacteria: *Escherichia coli*, which is a floral member of the colon. Other common urinary tract infection pathogens include enterobacterial strains (*Klebsiella spp*., *Enterobacter spp*. and *Proteus mirabilis*), and staphylococci and *Pseudomonas aeruginosa* can be isolated [6].

All bacteriuria in pregnancy should be treated and antimicrobial choices should reflect safety for both the mother and the fetus [7]. In simple urinary infection, nitrofurans and trimethoprim are antibiotics of first choice, and fosfomycin and β-lactamins are a second-line of treatment. In outpatient practice, fluoroquinolones are normally reserved for the treatment of complicated urinary tract infections. These medicines are considered safe and effective for the mother and the fetus during pregnancy [8,9]; however, recent research studies have demonstrated increasing resistance to these drugs [10,11]. Currently, the evolution and spread of antibiotic resistance bacteria is of great concern to the global health community [12]. To overcome this issue, scientists are searching for alternative efficient antimicrobial agents. The use of traditional medicine to treat a number of health problems (i.e., infections) in a natural way has been applied since ancient times. Honey bee (*Apis mellifera*) is one of the oldest traditional medicines, considered to be a traditional remedy to treat burns, infected and non-healing wounds and ulcers, boils, pilonidal sinus, venous and diabetic foot ulcers, and it has been shown that honey bee is capable of clearing infection from the wound and improving tissue healing. In addition, honey bee may possess anti-inflammatory activity and stimulate immune responses within a wound. The overall effect is to reduce infection and to enhance wound healing in burns, ulcers and other cutaneous wounds [13,14].

Honey bee contains about 200 substances, including amino acids, vitamins, minerals and enzymes, but it primarily contains sugar and water. The main carbohydrate constituents of honey are fructose and glucose [15].

The potential antibacterial agent in honey bee exhibits inhibitory effects towards approximately 60 bacterial species causing infections in the human body; including aerobes and anaerobes, Gram-positive and Gram-negative bacteria [16]. Different factors are responsible for the antimicrobial activity of honey, which include its sugar content, which is high enough to hinder microbial growth. This is believed to be a result of its osmotic effect, which prevents the growth of bacteria and therefore promotes healing. Honey bee is hygroscopic, meaning that it draws moisture out of the environment and dehydrates the bacteria with the aid of its hyperosmolar properties [17]. The low pH level of honey (mean 4.4) is unsuitable for bacterial growth and it can reduce wound colonization or infection. In addition, the antibacterial activity of honey bee may be due to hydrogen peroxide activity, which is continuously produced by the action of glucose oxidase; it is able to interact with bacterial cell proliferative signals, and thus affects bacterial growth even when honey is diluted [16,17,18]. Some floral sources such as flavonoids and aromatic acids provide additional antibacterial components in honey bee [19].

The Algerian populations have used honey bee frequently as a cure for several diseases. It is worth mentioning here that the spread of the use of honey in traditional and modern medicine has origins linked to the religious beliefs of Muslim people, where many Quranic and Islamic texts reveal that honey is a proven remedy [20].

The antibacterial activity of honeys from different countries has been extensively studied [21]. However, to the best of our knowledge, this is the first study that reported the clear effect of honey against many pathogens isolated from urinary tract infection during pregnancy. It aimed to evaluate the antibacterial activity of honey bee against the antibiotic-resistant bacteria isolated from the urine of pregnant women suffering from urinary tract infections.

## 2. Materials and Methods

### 2.1. Honey Bee Samples

**Figure 1:**
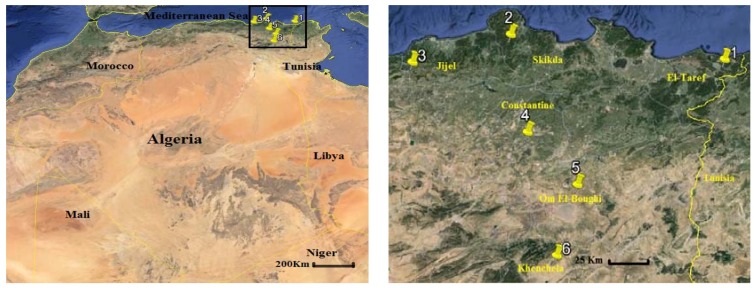
Geographic locations of the study area including honey harvesting sites.

Honey samples of the current study were collected by farmers from six localities in the east of Algeria during 2017. A map indicating the locations of the honey samples is shown in Figure 1. Six freshly harvested, natural, untreated and unpasteurized honey samples were used. Each sample was collected in a sterile universal container and kept at 4 °C in the dark until usage.

The following honey concentrations were prepared in sterile saline solution: 2.5%, 5%, 10%, 20%, 40%, 80% (*w/v*) and undiluted honey. Each honey sample was filtered through a 0.22 μm filter (Millipore, Nunc, Paramus, NJ, USA).

### 2.2. Strains

Honey samples were screened for their antibacterial activity against 11 bacterial strains isolated from the urine of pregnant women suffering from urinary infection at Ibn Rochd Hospital, Annaba, situated in east of Algeria. These strains are as follows: *Escherichia coli*, *Enterobacter aerogenes, Klebsiellaoxytoca, Klebsiella pneumoniae, Proteus mirabilis*, *Proteus vulgaris*, *Citrobacter koseri*, *Pseudomonas aeruginosa*, *Enterococcus faecalis, Staphylococcus aureus* and *Staphylococcus saprophyticus*. All tested strains were identified by conventional methods of microbiology (Gram staining, oxidase and catalase test, analytical profile index (API) 20E, API 20NE, API STAPH and API 20 STREP) (Biomerieux, Paris, France).

An inoculum of each strain was prepared, and the turbidity of the suspension was adjusted to achieve 0.5 McFarland (equivalent to that of 1.5 × 10^8^ colony-forming units (CFU)/mL) with the absorbance range of 0.08 to 0.1 by UV-Vis spectrophotometer at wave length of 620 nm.

All bacterial strains were subjected to antibiotic sensitivity tests by the Kirby Bauer’s disc diffusion method according to the Clinical and Laboratory Standards Institute [22] using Mueller Hinton agar medium (Biomerieux, Paris, France). The tested strains were selected because they are resistant to antibiotics used in the treatment of urinary infection during pregnancy.

### 2.3. Honey Samples Analysis

#### 2.3.1. Sensory Analysis

Sensory analysis is a tool to evaluate color and flavors of honey in order to understand if there is a relationship between the color, flavor and antibacterial activity of different honey samples. Sensory analysis is achieved according to the methodology described previously [23]. The samples were tested by a panel of 20 assessors that evaluate the taste and the color of honey samples.

#### 2.3.2. pH Measurement

The pH measurement was carried out by a pH meter (HI 98127, Hanna instruments) of a 50% (*w/v*) solution of each honey sample.

#### 2.3.3. Color Intensity

The absorbance of the honey samples was determined by the method of Beretta et al. [24]. The honey samples were diluted to 50% (*w/v*) with warm water (45–50 °C) and the solution was filtered using a 0.45 μm filter to eliminate large particles. The absorbance was measured using a UV-Vis spectrophotometer (T80 UV/VIS, PG instrument, England), at 450 and 720 nm and the difference in absorbance was expressed as mAU.

### 2.4. Antibacterial Activity

#### 2.4.1. Wells Assay

Wells (6 mm of diameter) were prepared in Mueller Hinton agar plates: these plates were inoculated by bacterial suspension and 150 µL of tested honey was added to each well. A hole filled with sterile water served as control. Plates were incubated at 37°C for 24h. The antibacterial activity of the samples was compared on the basis of the radius of a clear inhibition zone around the wells. The results are shown as mean values from triplicate measurements.

#### 2.4.2. Spectrophotometric Assay for MIC Determination

Minimum inhibitory concentrations (MICs) were determined using test described previously by Patton et al. [25]. A volume of 0.5 mL of standardized culture was added to 4.5 mL of tested honey, at each of the concentrations stated above. Control tubes containing broth only (negative or sterility control) or bacteria and broth (positive control) were used. Tubes were incubated in the dark at 37°C with shaking at 150 rpm for 24 h. The optical density was determined just prior to incubation (T_0_) and again after 24h incubation (T_24_) at 620 nm. The percent inhibition of growth was thus determined using the formula: percent inhibition (%) = 1− (OD test/OD of corresponding control) × 100. The MIC is reported as the lowest concentration of honey which results in 100% inhibition of growth of the test bacteria.

#### 2.4.3. Minimum Bactericidal Concentration Determination

The minimum bactericidal concentration (MBC) values were read as the lowest concentration of honey required for a 99.9% reduction in the viable strains. For determining MBC values, an aliquot (0.1 mL) of MIC mixtures that showed no growth was inoculated onto Mueller Hinton plates and incubated at 37 °C for 24 h.

#### 2.4.4. Time-Kill Assay

The time-kill assays were performed according to the method described previously [26]. 2 mL of honey was taken in sterile test tubes, one tube was inoculated with 20 μL of broth culture of the test bacterium in an initial concentration of approximately 10^7^ CFU/mL and the other tube was not inoculated (control). The tubes were incubated at 37°C with constant stirring (200rpm). Broth aliquots were collected at different time points, serially diluted in saline solution, plated on nutrient agar media and grown for 24h at 37 °C to determine the total CFUs in each tube.

### 2.5. Statistical Analysis

The results are reported as mean ± standard deviation (m ± SD). Multiple comparisons of means (Tukey HSD tests) were performed after each analysis of variance (ANOVA) to distinguish homogeneous groups among six origins using the software GraphPad Prism version 5 (Trial), Mars 12(2007).

## 3. Results

### 3.1. Antibiotic Sensitivity

The results of the susceptibility of urinary infection strains to prescribed antibiotics are summarised in Table 1. The results showed that all tested bacteria exhibited varying degrees of multidrug resistance of standard antibiotics used in urinary infections. The uropathogenic strains revealed the presence of high levels of multiple antimicrobial resistances.

### 3.2. Honey Analysis

The taste, pH values and color intensity of honey samples are reported in Table 2. Sample 2 has a bitter taste, whereas other samples have a sweet taste. As shown, all tested honey samples were acidic; the pH values ranged from 3.19 to 4.54. The color intensity of honey samples varied from 352 to 982 mAU for the light honey samples and from 1654 to 1965 mAU for the dark honey samples.

### 3.3. Inhibitory Diameters Determination

The results of inhibitory diameters of all honey bee samples are shown in Table 3. All tested honeys had a measurable antibacterial activity against all tested bacteria; the diameters of inhibition ranged from 19.67 to 53.33 mm. The results demonstrated that all honey samples had similar antibacterial activities (*p* > 0.05). However, there were highly significant differences for the susceptibility of different strains (*p* < 0.001): the inhibitory diameters of *P. aeruginosa* strain were less than other strains, which ranged from 19.67 to 27.33 mm. However, Gram-positive bacteria had the most important inhibitory diameters, ranging from 43.33 to 53.33 mm.

### 3.4. Determination of MICs and MBCs

From Table 4, it can be seen that the growth of all tested bacteria was inhibited by different samples of honey at concentrations of 5 to 20% (*w/v*), except *P. aeruginosa,* which has an MIC ranging from 20 to 40% (*w/v*) and an MBC ranging from 20 to 80% (*w/v*). However, Gram-positive bacteria were inhibited by all tested honey bee at concentration ranged from 2.5% to 5% (*w/v*).

### 3.5. Time Kill Curve

**Figure 2:**
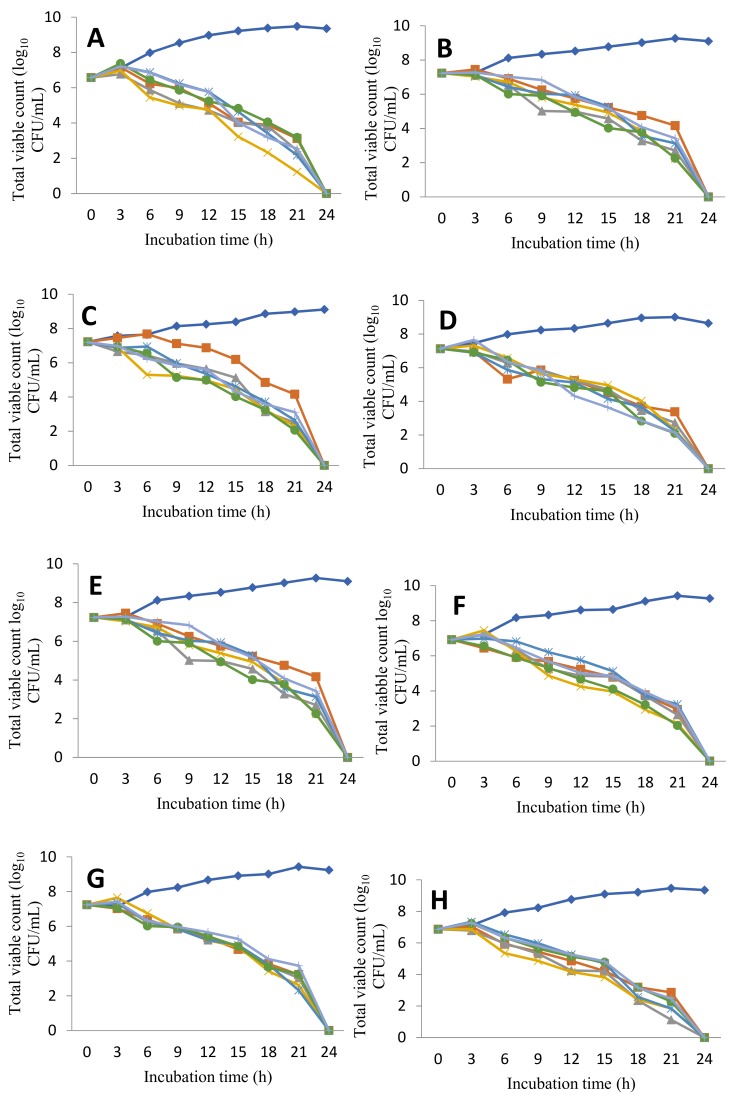

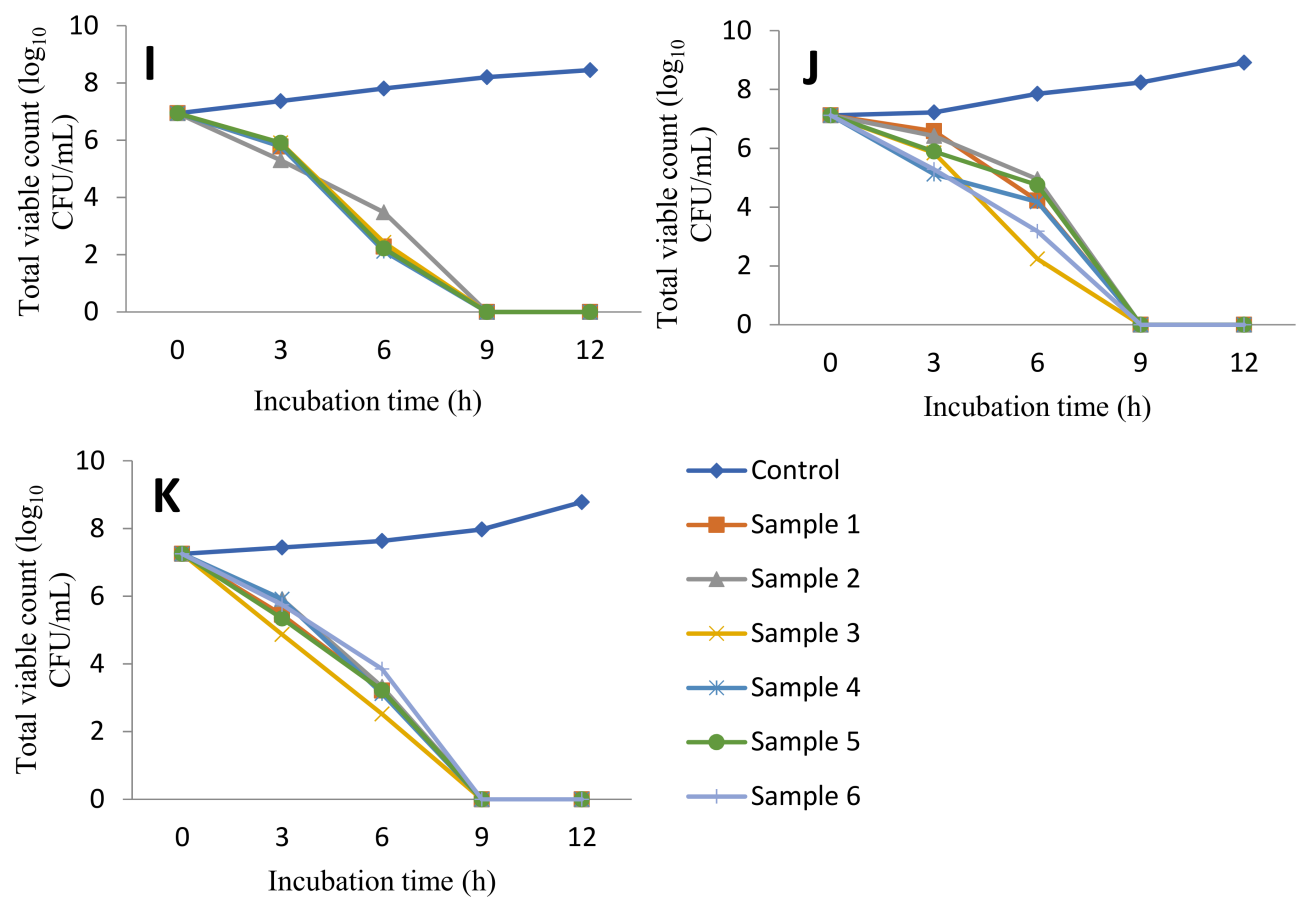
Time-kill curve showing *in vitro* bactericidal effect of six Algerian honey bee samples on multidrug resistant bacteria causing urinary tract infection during pregnancy. (**A**) *E. coli*, (**B**) *E.aerogens*, (**C**) *K. oxytoca*, (**D**) *K. pneumoniae*, (**E**) *P. mirabilis*, (**F**) *P. vulgaris*, (**G**) *C. koseri*, (**H**) *P. aeruginosa*, (**I**) *S. aureus*, (**J**) *S. saprophyticus*, (**K**) *E.faecalis.*

The results of the time-kill curve are indicated in Figure 2. The bactericidal action of honey bee seems to be different according to the bacterial strain; some strains were destroyed after nine hours of incubation (Gram-positive bacteria), but others were destroyed by the same sample of honey bee after 24 h.

## 4. Discussion

Antibacterial susceptibility testing, as illustrated in Table 1, revealed that all tested strains exhibited a high level of resistance to standard antibiotics used in urinary tract infections; similar results were previously reported [9]. However, some strains had poor sensitivity to gentamycin (45.4%) and to flouroquinolones (45.5%). Nevertheless, widespread usage may lead to resistance against these antibiotics. There have been no large-scale studies of the safety or risk of antibiotic use during pregnancy. However, the use of sulfonamide, trimethoprim and nitrofuran is known to increase the risk of neural-tube defects, cardiovascular defects, oral defects and urinary tract defects of birth [27,28]. Indeed, the use of aminoglycoside is known to be nephrotoxic to the fetus and should therefore be avoided during pregnancy. In addition, the use of macrolides (excluding erythromycin), quinolones, tetracyclines, during early pregnancy was associated with an increased risk of spontaneous abortion [29,30]. Thus, there is an urgent requirement for the development of new drugs constituting an effective and safe treatment which are not dangerous for both mother and fetus.

From Table 2, the analysis of honey samples showed that all of the tested Algerian honey samples were acidic in nature, with pH values that varied between 3.19 and 4.54. It was reported previously that the pH values of Algerian honey ranged from 3.70 to 4.00 [31] and 3.96 to 4.34 [32]. The acidity of any honey is directly related to the floral sources that created it. Honey bee contains a number of different acids, including about 18 amino acids, many different organic acids, as well as aliphatic and aromatic acids. The aromatic acids greatly contribute to the flavor of honey bee.

The absorbance of 50% (*w/v*) honey solutions was varied from 352 to 982 mAU for the light honeys and from 1654 to 1965 mAU for the dark and brown honeys. Honey samples from other countries were reported to have absorbance values between 25 and 3413 mAU in Italian honey [24], between 254 and 2034 mAU in Bangladeshi honey [33] and between 524–1678 mAU in Indian honeys [34]. This marked difference of color intensity might be a reliable index of the presence of pigments with antioxidant activities, such as carotenoids and some flavonoids, which are known to have antioxidant properties [35].

All honey samples have a sweet taste except Sample 2, which exhibited the highest color intensity, and has a bitter taste. The differing taste and color of honey bee directly depend on the flower foraged by the bee. There are a huge variety of flavors and colors in honey bee, depending on its origin.

The analysis of the diameter of inhibition values (in Table 3) showed that all tested honeys had potent antibacterial activity against all examined bacteria, and there highly significant differences for the susceptibility of different strains (*p*<0.001). As shown in Table 4, the diameters of inhibition varied between 28.33 and 37.33 mm and MICs varied between 5 and 20% in Gram-negative bacteria; except *P. aeruginosa*, which has inhibitory diameters ranging from 19.67 to 27.33 mm, however, the MIC and MBC values for *P. aeruginosa* were the highest, between 20 and 80% (*w/v*). These results are in agreement with those of Al-Nahari et *al*., who found that at concentrations of 20% and 50%, all types of honey had an effect on *P. aeruginosa* strains [36]. This bacterium is recognized for its multidrug resistance and it demonstrates practically all known enzymic and mutational mechanisms of bacterial resistance. The presence of efflux pumps reduces the effect of several antibacterial agents. Nonetheless, bacterial resistance to honey bee has never been reported, because its antibacterial activity is relate to a combination of components that may act in a synergistic manner to compromise the resistance [12].

Gram-positive bacteria were found to be more susceptible as compared to Gram-negative bacteria; the diameters of inhibition range from 43.33 to 53.33 mm, and MIC and MBC values range from 2.5% to 5% (*w/v*). These results are in agreement with a previous study [36]; however, opposite results were found by Al-Namma, (2009) which showed that honey bee is more active against Gram-negative bacteria [37].

Moreover, in order to assess the effect of honey samples on bacterial cell viability, the time-kill curves as illustrated in Figure 2 demonstrated that the treatment of bacterial strains with honey bee samples was successful in killing within nine to twenty-four hours. The bactericidal action of honey bee seems to be different according to the bacterial strain. Generally, Gram-positive bacteria are more sensitive to biocides than Gram-negative bacteria: this is related probably to the composition of the cell envelope. The cell wall of Gram positive bacteria lacks an outer lipopolysaccharide membrane but has a thick layer of peptidoglycan, which forms a thick fibrous layer. This facilitates access of cell-wall active biocides to their site of action [38,39]. In Gram-negative bacteria, honey or any other biocide (such as essential oil) has to traverse the lipopolysaccharide layer which provides a barrier that allows Gram-negative bacteria to be more resistant to honey bee. Efflux proteins may also be present in Gram-negative cell walls. These are relevant to biocides that act intracellularly and pump them out of the cell, lowering the intracellular honey concentration to a level where it becomes less active or inactive [38,39]. Phenolic compounds, which are also present in honey, generally show antimicrobial activity against Gram-positive bacteria. Their effect depends on the amount of the compound present; at low concentrations, they can interfere with enzymes involved in the production of energy and at higher concentrations, they can denature proteins [38].

The higher potency of the Algerian honey might be related to the high values of total polyphenol and flavonoid contents in this region, which seem to influence the honey’s antimicrobial activity.

## 5. Conclusions

The main results of the present work showed that the six used honey bee samples had a similar antibacterial activity against all the 11 examined strains. Furthermore, the bactericidal action efficiency of honey bee was different according to the bacterial strain, where Gram-positive bacteria were found to be more susceptible as compared to Gram-negative bacteria. Therefore, Algerian honey bee can be a promising alternative that might substitute antibiotics used in the treatment of urinary infections in pregnant women; this choice constituted an effective and safe treatment for both the mother and the fetus. Indeed, microbial resistance to honey has never been reported, because its antibacterial activity is related to the complex composition of honey bee, which contains a combination of components that may act in a synergistic manner to compromise the resistance; this makes honey bee a very promising topical antimicrobial agent against the infection of antibiotic-resistant bacteria.

## Figures and Tables

**Table 1:** Susceptibility of urinary infection strains to prescribed antibiotics.

Strain	Susceptibility to Prescribed Antibiotics												
	β-Lactams			Cephalosporin		Fluoroquinolone			Aminglycosides		Others		
	OX	AM	AMC	CN	CF	OF	NA	CIP	GM	TM	NIT	FOS	SXT
*E. coli*	ND	R	R	R	R	S	S	S	S	R	R	S	R
*E. aerogenes*	ND	R	R	R	R	S	S	S	R	R	R	R	R
*K. oxytoca*	ND	R	R	R	R	S	S	S	S	R	R	R	R
*K. pneumoniae*	ND	R	R	R	R	R	R	R	R	R	R	R	R
*P. mirabilis*	ND	R	R	R	R	R	R	R	R	R	R	R	R
*P. vulgaris*	ND	R	R	R	R	R	R	R	R	R	R	R	R
*C. koseri*	ND	R	R	R	R	S	S	S	S	R	R	R	R
*P. aeruginosa*	ND	R	R	R	R	R	R	R	R	R	R	R	R
*S. aureus*	R	ND	ND	R	R	R	R	R	S	R	R	R	R
*S. saprophyticus*	S	ND	ND	R	R	S	S	S	S	R	R	S	R
*E. faecalis*	R	ND	ND	R	R	R	R	R	R	R	R	R	R
Susceptibility Percentage (%)	33.3	0	0	0	0	45.4	45.4	45.4	45.4	0	0	18.2	0

OX: oxacilline, AM: amoxicillin, AMC: amoxicillin–clavulanic-acid, CN: cefazolin, CF: cefexim, OF: ofloxacin, NA: nalidixicacid, CIP: ciplofloxacin, GM: gentamicine, TM: tobramicine, NIT: nitrofurantoїne, FOS: fosfomicine, SXT: sulfamethoxazol-trimethoprim, R: resistant, S:susceptible, ND: not determined.

**Table 2:** Taste, pH and color intensity of honey samples.

Honey Sample	Taste	pH	Color	Color Intensity (mAU)
Sample 1	Sweet	3.44	Light brown	742
Sample 2	Bitter	4.54	Dark brown	1965
Sample 3	Sweet	3.86	Brown	829
Sample 4	Sweet	3.19	Dark brown	1654
Sample 5	Sweet	3.54	Brown	982
Sample 6	Sweet	4.02	Light brown	352

**Table 3:** Mean diameter (mm) of inhibition by honey samples against uropathogenic strains.

Strain	Diameter of Inhibition (mm±SD)					
	Sample 1	Sample 2	Sample 3	Sample 4	Sample 5	Sample 6
*E. coli*	30.33 ± 1.56	32 ± 0.67	36.67 ± 1.11	33.67 ± 0.44	30.00 ± 0.67	30.00 ± 0.67
*E. aerogenes*	30.33 ± 1.11	31.33 ± 0.89	37.00 ± 0.67	33.67 ± 0.89	31.33 ± 1.11	31.00 ± 0.67
*K. oxytoca*	28.33 ± 1.11	31.33 ± 0.44	36.00 ± 1.33	33.33 ± 0.44	29.00 ± 1.33	30.67 ± 1.56
*K. pneumoniae*	29.67 ± 1.11	32.33 ± 0.44	37.33 ± 0.89	35.00 ± 0.67	29.33 ± 0.44	29.33 ± 0.44
*P. mirabilis*	30.00 ± 0.67	31.33 ± 0.89	36.33 ± 1.11	33.33 ± 1.11	30.33 ± 0.44	28.67 ± 0.89
*P. vulgaris*	30.33 ± 1.11	32.33 ± 0.44	35.67 ± 1.56	31.00 ± 0.67	29.67 ± 0.44	29.67 ± 0.89
*C. koseri*	29.00 ± 1.33	30.33 ± 0.44	34.67 ± 0.89	31.67 ± 1.56	29.00 ± 0.67	29.00 ± 0.67
*P. aeruginosa*	19.67 ± 1.56	24.33 ± 0.89	27.33 ± 0.89	26.00 ± 0.67	20.33 ± 0.89	20.33 ± 0.44
*S. aureus*	47.667 ± 1.11	48.67 ± 0.89	50.67 ± 1.11	49.67 ± 0.44	47.67 ± 0.44	47.33 ± 1.56
*S. saprophyticus*	48.33 ± 0.89	50.00 ± 0.67	53.33 ± 0.44	50.33 ± 0.89	47.67 ± 0.44	47.67 ± 0.89
*E. faecalis*	43.67 ± 0.89	44.00 ± 0.67	46.67 ± 0.44	44.00 ± 0.67	43.33 ± 0.89	43.67 ± 0.44

**Table 4:** Minimum inhibitory concentration (MIC) and minimum bactericidal concentration (MBC) values of honey samples against uropathogen strains (% *w/v*).

Strain	Sample 1		Sample 2		Sample 3		Sample 4		Sample 5		Sample 6	
	MIC	MBC	MIC	MBC	MIC	MBC	MIC	MBC	MIC	MBC	MIC	MBC
*E. coli*	10	10	10	10	5	10	10	10	10	10	10	10
*E. aerogenes*	10	10	10	10	5	10	10	10	10	10	10	10
*K. oxytoca*	20	20	10	20	5	20	10	20	20	20	10	20
*K. pneumoniae*	20	20	10	20	5	20	5	20	20	20	20	20
*P. mirabilis*	10	10	10	10	5	10	10	10	10	10	20	10
*P. vulgaris*	10	10	10	10	5	10	10	10	20	10	20	10
*C. koseri*	20	40	10	40	5	40	10	40	20	40	20	40
*P. aeruginosa*	40	80	20	40	20	40	20	20	40	80	40	80
*S. aureus*	2.5	2.5	2.5	2.5	2.5	2.5	2.5	2.5	2.5	2.5	2.5	2.5
*S. saprophyticus*	2.5	2.5	2.5	2.5	2.5	2.5	2.5	2.5	2.5	2.5	2.5	2.5
*E. faecalis*	5	5	5	5	2.5	5	5	5	5	5	5	5
